# Dermoscopy of Type 1 Lepra Reaction in Skin of Color

**DOI:** 10.5826/dpc.1003a83

**Published:** 2020-06-29

**Authors:** Abhijeet Kumar Jha, M.D. Zeeshan, Pankaj Tiwary, Anupama Singh, Prasoon Kumar Roy, R.K.P. Chaudhary

**Affiliations:** 1 Department of Skin & V.D., Patna Medical College and Hospital, Patna, Bihar, India

**Keywords:** leprosy, type 1 lepra reaction, dermoscopy in skin of color, erythema nodosum leprosum, dermoscopy

## Introduction

Leprosy reactions are immunological reactions due to the changes in a patient’s immune status in response to *Mycobacterium leprae* that may occur before, during, or even after the completion of multidrug therapy. Leprosy reactions are divided into 2 types. Type 1 lepra reaction (reversal reaction) is characterized by the development of acute erythema and swelling of existing skin lesions or by the appearance of new lesions and/or neuritis [1,2]. Type 2 lepra reaction (erythema nodosum leprosum) is the appearance of skin nodules due to the immune complex–mediated complication of leprosy [3].

T1R is a type IV hypersensitivity reaction that occurs mostly in borderline leprosy patients [4]. Clinically, T1R may present with similar morphology to other granulomatous skin conditions such as lupus vulgaris (LV), sarcoidosis, and granuloma faciale. As we know, dermoscopy can be used to facilitate the differential diagnosis of granulomatous skin conditions [5]. The present study aims to describe dermoscopic patterns in T1R according to the severity of lesions and the type of leprosy.

## Case Presentation

**Figure 1:**
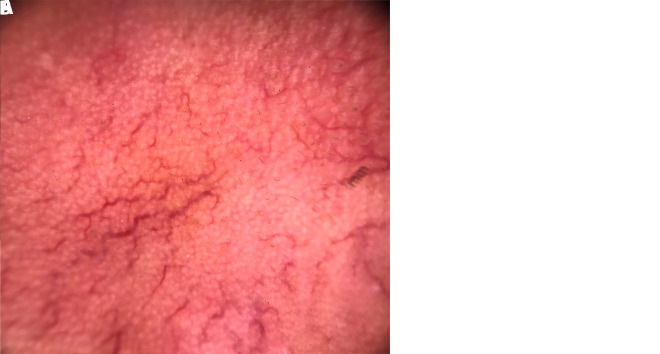
(A) Erythematous plaque on the face. (B) Dermoscopy (polarized, ×10) showing yellowish orange area with arborizing vessels.

**Figure 2:**
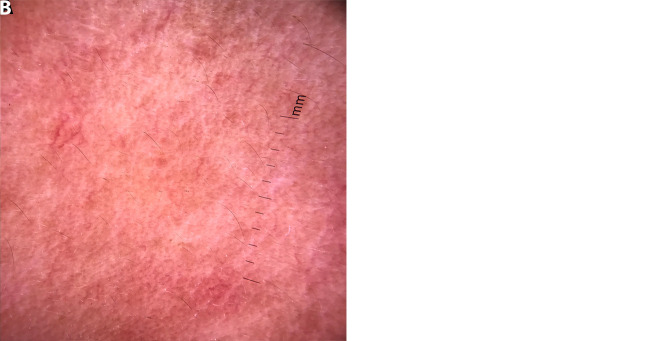
(A) Erythematous plaque on the face. (B) Dermoscopy (polarized, ×10) showing reddish yellow area with linear vessels.

**Figure 3:**
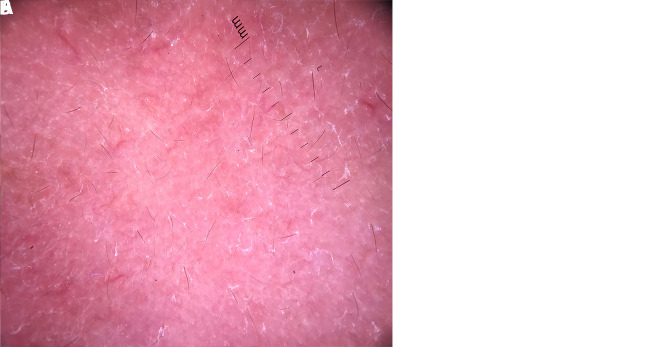
(A) Erythematous plaque on the face. (B) Reddish areas along with white structureless areas with fine short linear blurry vessels.

The present work was designed as a prospective, tertiary urban hospital–based, observational study during the period from August 2016 to January 2017. Institutional ethical clearance was obtained, and patients clinically and histopathologically diagnosed as having T1R were included. Leprosy patients were classified using the Ridley-Jopling classification [1]. Dermoscopy was performed by 2 independent dermoscopists (experience 7 years and 5 years, respectively). Dermoscopy (polarized, ×10) was done using DermliteDL4 (3Gen, San Juan Capistrano, CA), and photographs were captured by Apple iPhone 7 from the same site where biopsy was done (facial lesion)and sent for histopathology. Dermoscopic features for lepra diagnosis included background color and type of vessels. A total of 14 patients with type 1 reaction were included in the study. Type 1 lepra reaction was seen mostly in patients who were treated with multidrug therapy for leprosy for less than 6 months. Among the 14 cases, 8 patients (57.14%) were previously diagnosed as having borderline tuberculoid leprosy (Figures 1A, 2A, 3A) and 6 cases (42.85%) as having borderline lepromatous leprosy. Nine male (64.28%) and 5 female patients (35.71%) were included, with age ranging from 21 to 47 years. At our dermoscopic examination, yellowish orange areas were observed in 6 cases (42.85%) and particularly reddish orange areas were seen in 8 cases (57.14%); arborizing vessels (Figure 1B), fine short linear vessels (Figure 2B), and linear blurry vessels (Figure 3B) were detected in 10 cases (71.42%) and in 4 cases (28.57%), respectively; white scales were present in 1 case (7.14%).

## Conclusions

Dermoscopy is a noninvasive tool widely used in the diagnosis of skin tumors and inflammatory skin disorders [6]. It is also helpful in assessment of vascular structures and color variations, which are not clinically visible to the naked eye. Thus, dermoscopy may be considered between clinical examination and histopathology [7].

In the study conducted by Ankad and Sakhare [8], the dermoscopic features of the patients with borderline tuberculoid leprosy characteristically showed white areas, yellow globules, and linear branching telangiectasia. In our study, patients with borderline tuberculoid leprosy with severe T1R [8] showed reddish background and white structureless areas with fine short linear blurry vessels, probably due to an increased number of lymphocytes and loss of normal granuloma organization. Yellow globules were seen in 6 cases of T1R. Orange-yellow globules with telangiectasia are generally considered to be the hallmark of dermal granulomas. However, yellow translucent globules with branching telangiectasia could be also seen in LV and sarcoidosis [9].

The vessels in T1R were mostly blurry in contrast to sarcoidosis and LV, where the vessels appear usually quite sharp. This may be due to the fact that in sarcoidosis and LV, the granulomatous infiltrates are dense, thus pushing the vessels upward, closer to the surface with a consequent sharper appearance [10]. In conclusion, dermoscopic features such as linear blurry vessels within reddish or orangish areas could typify T1R.
